# Data-Driven Surveillance: Effective Collection, Integration, and Interpretation of Data to Support Decision Making

**DOI:** 10.3389/fvets.2021.633977

**Published:** 2021-03-12

**Authors:** Fernanda C. Dórea, Crawford W. Revie

**Affiliations:** ^1^Department of Disease Control and Epidemiology, National Veterinary Institute, Uppsala, Sweden; ^2^Computer and Information Sciences, University of Strathclyde, Glasgow, United Kingdom

**Keywords:** epidemiology, machine learning, big data, data analyses, linked data

## Abstract

The biggest change brought about by the “era of big data” to health in general, and epidemiology in particular, relates arguably not to the volume of data encountered, but to its variety. An increasing number of new data sources, including many not originally collected for health purposes, are now being used for epidemiological inference and contextualization. Combining evidence from multiple data sources presents significant challenges, but discussions around this subject often confuse issues of data access and privacy, with the actual technical challenges of data integration and interoperability. We review some of the opportunities for connecting data, generating information, and supporting decision-making across the increasingly complex “variety” dimension of data in population health, to enable data-driven surveillance to go beyond simple signal detection and support an expanded set of surveillance goals.

## Introduction

Increases in data volume, diversity and speed have affected all aspects of human life. As we advance into the 21st century, Simonsen et al. (1) highlight two main streams that are pushing health surveillance into the “Big Data Era”: the advancements in laboratorial detection tools which traditional surveillance rely on, and a dramatic increase in the number of health and non-health related data streams that can be exploited for surveillance. However, as Leyens et al. (2) point out, “the simple fact that there is more data is not useful to public health unless we are able to turn it into ‘actionable data' for improved health outcomes and more effective and efficient health systems.”

While health surveillance systems continue to adapt, improving traditional components [e.g., (2–4)] and adding others based on the exploitation of novel data streams [e.g., (5–8)], their progress fades in comparison to that seen in other sectors (1), from business and marketing to the more related area of diagnostic services within human health. While data scientists seem to agree that a significant big data trend in 2017 was an end to talk about it as if it were a novelty (9), in health surveillance “big data” remains a buzz word. A number of publications have discussed the challenges and potential benefits of incorporating big data into surveillance, but a framework for the operationalization of data-driven surveillance has seldom been discussed. Moreover, discussions around the exploitation of novel data streams has been focused almost exclusively on emergence prediction and early disease detection, in detriment of other surveillance goals, such as situational awareness for non-communicable and endemic diseases, and disease freedom demonstration. Based on the results of a workshop carried out in late 2017, and supported by a scoping review, we discuss the challenges and opportunities for implementing data-driven surveillance frameworks as a 3-step process: data integration; data processing to generate information; and making outputs from data analyses accessible and usable by decision-makers.

## Methods

On October 10th and 11th, 2017, the Uppsala Heath Summit gathered around 200 delegates from different sectors, and from around the world, to discuss priorities for preventing, detecting and responding to infectious disease threats using a One Health approach (10). A dedicated 3 h workshop was conducted by the authors to explore the theme of innovation and big data in health surveillance. The 63 workshop participants brainstormed to identify and prioritize opportunities to achieve data-driven decision-making in population health, within the One Health context. Participants came from a range of sectors: 16 were from universities, 11 from the private sector, 22 from governmental agencies and one from a global health organization. This was a multi-disciplinary group, from the fields of public health (11), animal health (12), pharmacovigilance (13), health and medicine (3), data science (4), climate (1), and geography (1). Most participants worked in European countries, with three participants from Africa, two from North America and one from South America. Informed by a literature search targeting articles in the health surveillance domain which used the term “big data,” workshop discussions were organized into four main groups of “big data analytics” (BDA) challenges: technical, operational, normative (cultural and ethical challenges), and funding. A summary of the workshop discussions, within the four main challenge themes, is already available in the post-conference report (10). Following the workshop, we have organized the discussion according to actual implementation steps, laying out a “data to actionable information” continuum, and enriched it with bibliography relevant for each section.

We have also updated and reviewed the literature search specifically targeting BDA. We searched Scopus for papers published up to December 2020 in the general area of health surveillance which contained the term “big data” [TITLE-ABS-KEY (“big data” AND surveillance AND (health OR disease OR syndromic))]. This search returned 492 papers. After reviewing title and abstract, and reading selected papers for which full-text was available in English, we selected a total of 47 papers which specifically discuss data science and data innovation challenges and opportunities in any area of health surveillance.

We have not cited all papers here due to space limitations, but the full list of 44 selected papers is available in the Supplementary Material, and also at (http://datadrivensurveillance.org/dds_ICAHS2020).

## Results and Discussion

### Step 1—Connecting Data

The most significant changes in the area of health data in general, and epidemiology in particular, arguably relate not to the volume of data, but to their variety. An increasing number of innovative data sources, including many not collected specifically for health purposes, can now be used for epidemiological inference and contextualization (14). The challenges of data integration have been discussed by many researchers (2, 12, 13, 15, 16). Often, however, the discussion confuses issues around data access and privacy, with the actual technical challenges of data integration and interoperability. The latter issues are central to contemporary surveillance, which increasingly relies on combining evidence from multiple data sources.

Surveillance data have traditionally been classified by mode of acquisition: active or passive. With the advent of “big data,” the concept of data acquisition becomes less central—we move from intentionally producing surveillance data, to taking advantage of ubiquitous data sources generated as a part of many processes, health related, or not (11). The technical challenge is no longer validating a dataset in which each observation was intentionally recorded, but rather mining data streams for valid evidence to support decision making (11).

**Figure 1:**
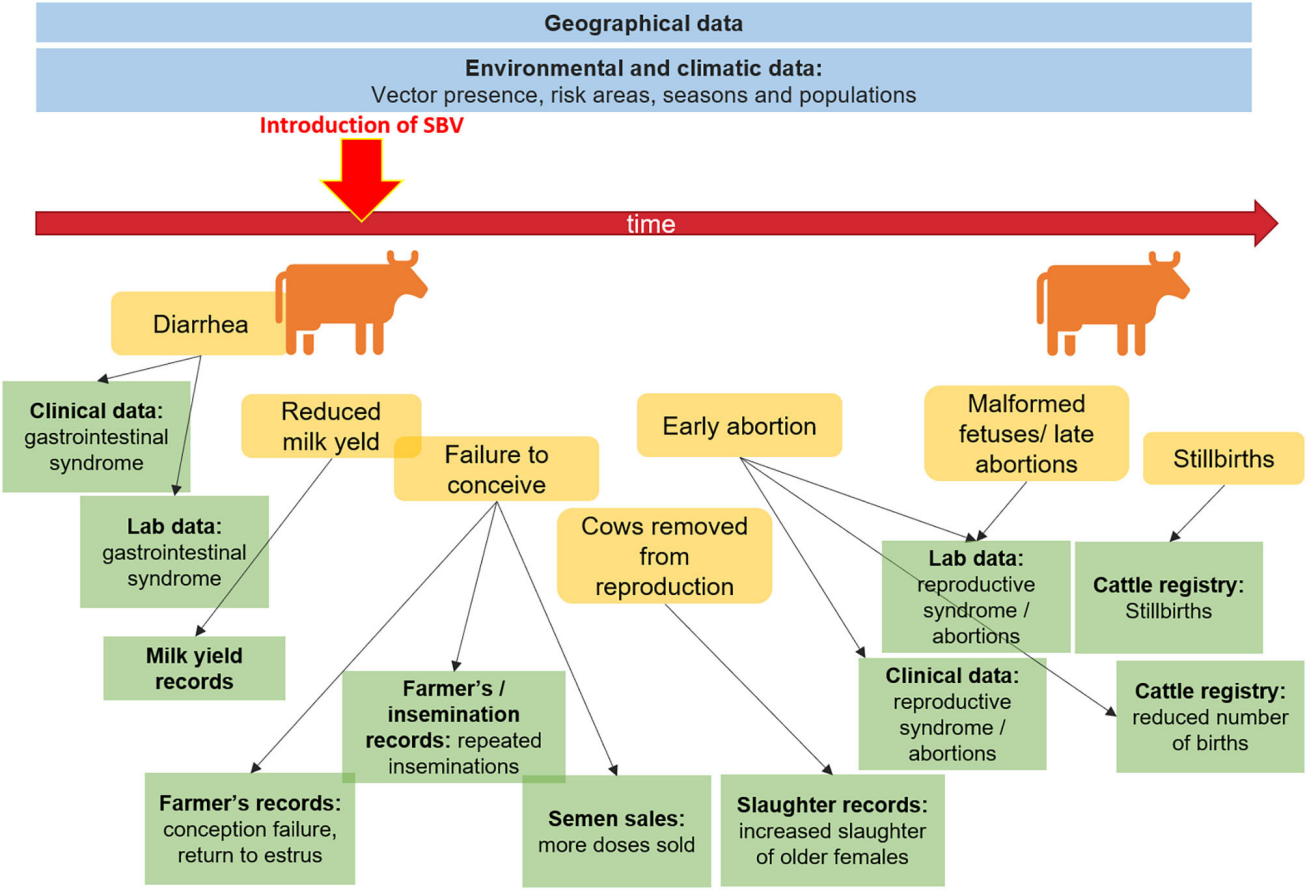
Potential data sources to aid surveillance before and after the introduction of Schmallenberg virus.

Figure 1 illustrates the potential data streams from which signals of a health hazard occurrence might originate for the case of Schmallenberg virus being introduced into a dairy herd. This figure represents a limited snapshot of the health continuum of interest for animal health. We can imagine the increased complexity involved if we were to consider a zoonotic pathogen, and had to factor in exposure to humans through the food production cycle, or environmental exposure. The variety of novel data streams that can support surveillance has been reviewed in detail for animal health (17, 18), drug safety and health care (2), food safety (19), and one medicine (20). Opportunities associated specifically with spatial data sources (21) and search query data (22) have also been reviewed.

In addition to the access and interpretation of a greater number of opportunistic data sources, there are also increased opportunities to redesign the purposeful collection of surveillance data in the digital era. Salathé (14) discussed applications to drug safety monitoring, while a broader review of crowd-sourcing, citizen sensing and sensor web technologies for health is given by Kamel Boulos et al. (23). Workshop participants highlighted, in particular, the use of apps for patient reporting or self- diagnosis, which can have value along the entire surveillance continuum: from prevention, to communication with the public during response.

The sources of data we have access to determine the types of evidence we can extract, and the timeliness of such extraction. As Han and Drake (24) note, our ability to move toward predictive capacity is limited not by technology, but by access to appropriate data. To achieve a paradigm shift in disease control, moving from disease response to disease intelligence, a resilient health system must be underpinned by environmental, geographic, and population data (2, 24).

During the workshop, the group concluded that the single biggest barrier to gaining insights from data, particularly in real-time settings, was data integration. The need to “break the barriers of siloed data” was often mentioned as a priority. Timely access to integrated data was considered the main challenge to using data-driven evidence in emergencies, such as during outbreak response.

The issue of data integration and interoperability (25) is particularly important when targeting long chains involving multiple actors, such as in food safety surveillance (19). The lack of standardized data was repeatedly mentioned as a barrier for data processing and interpretation. However, as the discussion around this issue matured, most participants agreed that it was unrealistic to expect data standardization, as in fact many standards already exist for health data, but are not used. Most importantly, many existing standards contribute only to achieving structural (*syntactic*) interoperability.

As the secondary use of data sources (re-use) increases, and models demand integration of data from multiple disciplines, we will increasingly require *semantic* interoperability. Semantic interoperability is concerned with ensuring that the integrity and meaning of the data is preserved throughout the integration process (26). This is achieved by storing data in machine interoperable formats making use of knowledge models that explicitly document, for humans and for machines, the domain knowledge and assumptions under which data were collected and are stored (27). Ontologies allow domain experts to create knowledge models that can be interpreted both by humans and machines (28). Using such models, computers can reason with data without relying on the use of specific codification. For an example in animal health, see Dórea et al. (29).

### Step 2—Generating Information

A common skepticism related to big data comes from authors who highlight its potential to become a “hypothesis generating machine,” capable of detecting correlation, but not causation (12, 30). The question should perhaps not be whether big data are useful, but what they are useful for. In surveillance, associations may be an important source of information for decision on interventions that aim at risk mitigation or case finding, even in the absence of any proven causal association. Iwashyna and Liu (11) point out that the questions which big data cannot answer are similar to those that are also a challenge in most observational studies, such as prescriptive questions. The authors suggest three main types of questions that can be addressed with big data: prognostic questions (what is going to happen), which “require temporally stable associations, not underlying causal models”; predictive questions (what will likely happen if something different is done); and patterning questions (describing population patterns).

Automated access to continuous streams of data has allowed monitoring of population patterns—and early detection of unexpected changes—at earlier and earlier steps on the disease continuum. From direct monitoring of early registers (e.g., veterinarian calls or visits to the emergency room), to even less specific, but earlier signs of health change, such as over-the-counter drug sales. This component has been coined “syndromic surveillance” due to the initial focus on the monitoring of unspecific clinical symptoms in public health (31). The methodology has been applied in animal health to a number of data sources that are not necessarily “syndromic” (32, 33), and its utility is being increasingly explored for situational awareness rather than simply for early disease detection. To that end, Smith et al. (34) reported the need to focus on system sustainability and usefulness as one of the main lessons learned from two decades experience with syndromic surveillance in the UK. They argued that systems should be designed with a focus on the uses, not the data sources, and should aim to serve multiple public health objectives.

For a more complete review of the architectures and specific methods for big data analysis in health surveillance, we refer readers to (19, 25, 35, 36). For a review of the use of terms “big data,” “informatics” and “bioinformatics” in the animal health and veterinary medical literature, we refer to (37).

During the workshop, the discussion focused not on what analysis tools to use, but on how to incorporate available methods within routine surveillance. The gap between technological and methodological innovation, as well as implementation in field settings are also discussed in (38). An important message related to the fact that surveillance officials should not only have access to the right tools, but should also be capable of using them effectively. “Efficient people and technology,” as one captured note summarized this point. The need for more training was repeatedly listed, in addition to the importance of making tools that are more accessible to domain experts; that is, user-friendly and available in local languages. Chiolero and Buckeridge (39) called these the “knowledge brokers” needed to “bridge data science, health monitoring and public health.” Reference was also made to the training needs discussion presented in Brownson et al. (40).

### Step 3—Supporting Decision-Making

**Figure 2:**
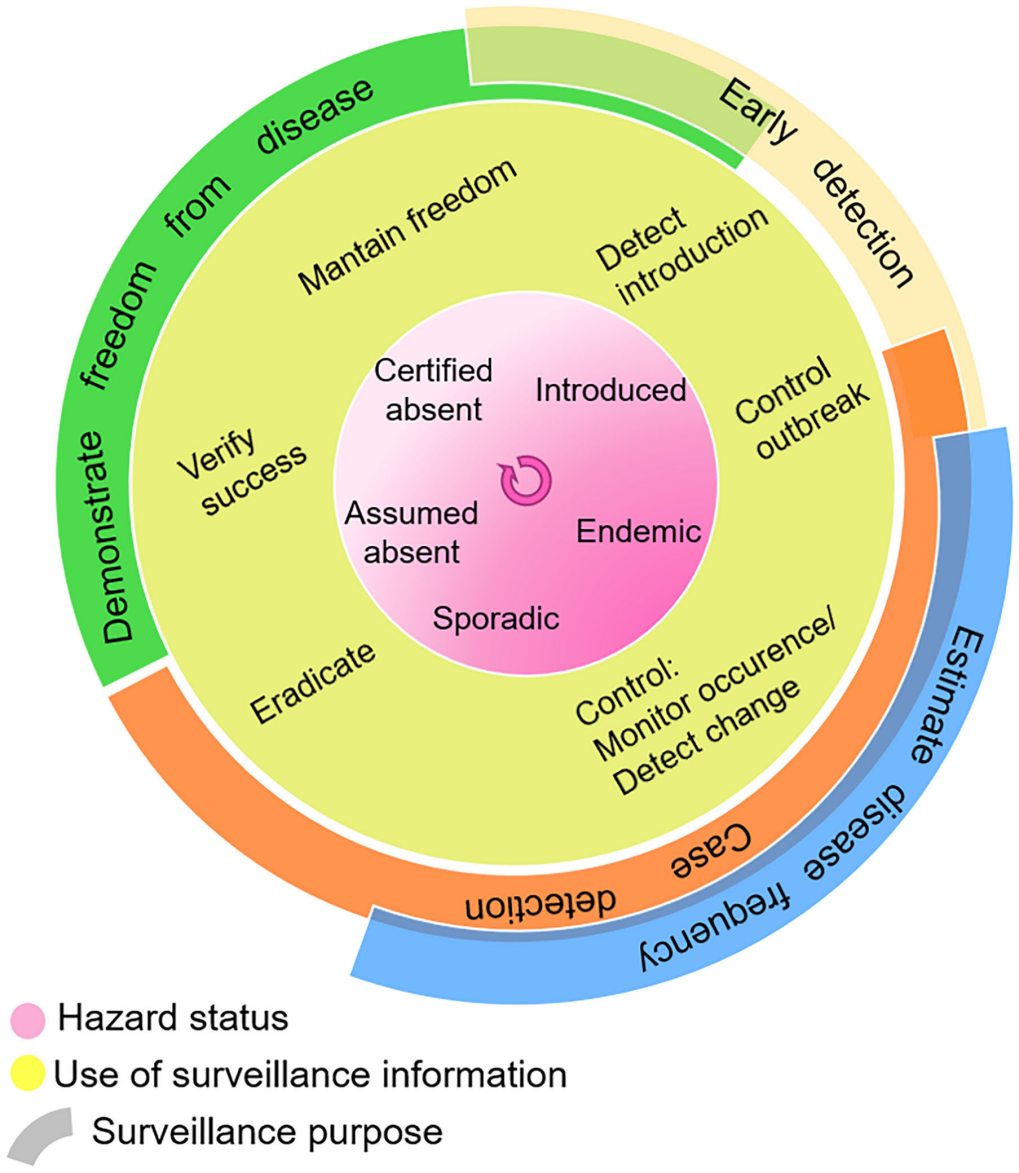
Use of surveillance information in the context of hazard status, and surveillance purpose. Adapted with permission from Linda Hoinville (41).

Surveillance activities are designed according to the desired use of surveillance information, as summarized in Figure 2. This in turn depends on the hazard occurrence in the target population or geographical area. As can also be seen in this figure, the boundaries are not always clear, and purposes can overlap. This highlights an overall workshop conclusion that the separation of surveillance goals may be artificial, and that a data-driven decision support system should be designed to strengthen all stages of disease control. Chiolero and Buckeridge (39) emphasize the role of decision-makers in identifying surveillance needs, setting priorities, and evaluating the effect of interventions. They added to their “glossary of public health surveillance in the era of big data” the idea of a continuum from data, to information, to *evidence* (which “emerges from the comparison of information”), then “used to build actionable knowledge” (DIEK pyramid) (42).

Increases in data variety and velocity have opened up new surveillance opportunities, most notably in relation to disease prevention and early detection. The ability to train statistical algorithms on a large quantity and variety of data to identify relationships and monitor interactions allows us to monitor risks in space and time [creating a “riskscape” (24)], and respond to these risks, rather than to occurrence. It creates the opportunity to improve timeliness and population coverage, and increase resolution (spatial and temporal) (25), leading to infectious disease intelligence—knowing what, when, why, and how to respond (24). In public health, the use of new data and technologies to assess population health with increased accuracy and granularity at temporal and geographical levels, delivering programs tailored to specific populations, has been coined “precision public health” (39, 43).

While the advent of “big data analysis” has been extensively discussed for disease prediction and early response, its support to other surveillance goals has often been overlooked. Access to digitalized and novel data streams can increase the timeliness of surveillance information, but can also “improve temporal or spatial resolution of surveillance, add surveillance to places with no existing systems,. measure aspects of a transmission/disease process not captured by traditional surveillance, and increase the population size under surveillance” (44). Antoine-Moussiaux et al. (45) argue that a focus on detection of disease signals may miss the true value of surveillance, which lies in its continuity. They propose that health surveillance should be viewed as an information system, which continuously provides feedback to inform the prioritization of actions.

**Figure 3:**
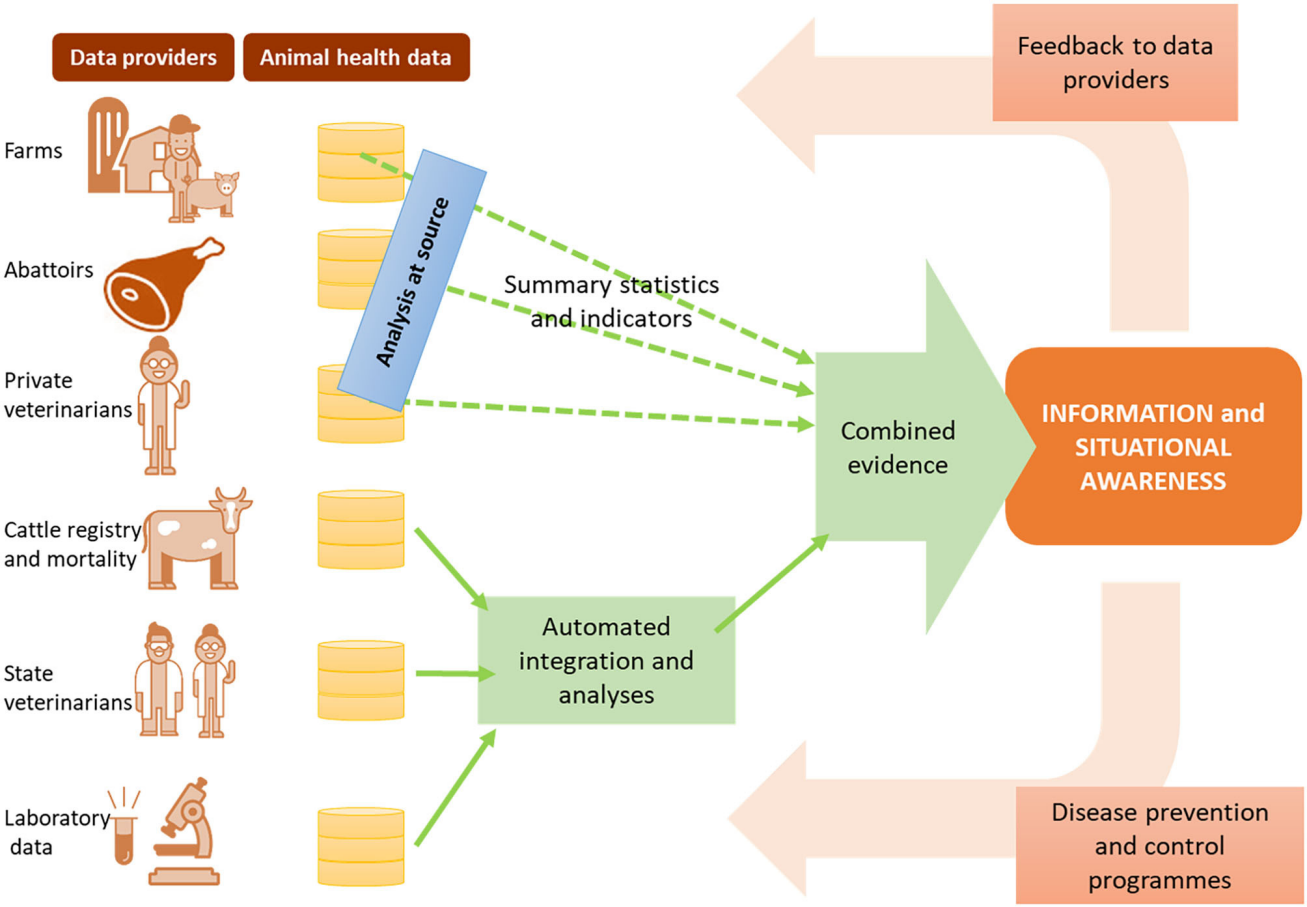
Data-driven surveillance framework being developed in Sweden, as an example of how information and evidence can be produced from multiple data sources without relying on data sharing.

This assumes we have addressed the two previous steps, and as such have access not simply to “big data,” but to FAIR data—findable, accessible, interoperable and reusable (46). In a scenario of semantically interoperable data we can more readily employ machines to reason over complex knowledge, and support surveillance decision-making holistically. Data variety and even issues of data accessibility are resolved, rather than being barriers. In an ongoing project in Sweden, for example (Figure 3), we are researching methods to combine evidence from analysis, rather than combining data directly. Data are analyzed at source, with signals being compiled centrally.

Moreover, a data-driven surveillance framework assumes that decision-makers have access to the outputs of big data analysis with the same level of “FAIRness” —this requires the availability of decision supporting dashboards that allow end users to query through the data sources in consumable formats, and navigate through the outputs of analysis in transparent ways. Most importantly, it requires that the value extracted from the data is returned to all relevant stakeholders (Figure 3), creating a positive cycle of encouragement not only for data accessibility, but also for data quality.

## Conclusion

Solving the technological barriers to extracting information from big data is only the first step toward a framework for evidence-based decision making. Data-driven support to surveillance in practice will depend on having access to the right data, employing the right methods, and making the outputs accessible and understandable to the right stakeholders. Participants in the workshop, as well as several papers reviewed (1, 14, 19, 47), highlighted that data-driven components could support traditional surveillance, but that the surveillance systems of the future will be a hybrid of traditional and data-driven methods. System design should focus on health surveillance goals and utility to the decision-makers. Information generation is data-driven, but system design should not be. Using novel data sources to complement those used traditionally will merge the best of both worlds—though gains in timeliness and predictive power will come at the cost of dealing with all of the complexity in these novel data sources (1).

## Data Availability Statement

The original contributions presented in the study are included in the article/Supplementary Material, further inquiries can be directed to the corresponding author/s.

## Author Contributions

All authors listed have made a substantial, direct and intellectual contribution to the work, and approved it for publication.

## Conflict of Interest

The authors declare that the research was conducted in the absence of any commercial or financial relationships that could be construed as a potential conflict of interest. The reviewers JA and JB declare a past co-authorship with one of the authors FD and state that the process nevertheless met the standards of a fair and objective review.
