# Corrigendum: Data-Driven Surveillance: Effective Collection, Integration, and Interpretation of Data to Support Decision Making

**DOI:** 10.3389/fvets.2021.789696

**Published:** 2021-11-01

**Authors:** Fernanda C. Dórea, Crawford W. Revie

**Affiliations:** ^1^Department of Disease Control and Epidemiology, National Veterinary Institute, Uppsala, Sweden; ^2^Computer and Information Sciences, University of Strathclyde, Glasgow, United Kingdom

**Keywords:** epidemiology, machine learning, big data, data analyses, linked data

In the original article, a potential conflict of interest was not declared. The corrected Conflict of Interest statement appears below.

“The reviewers JA and JB declare a past co-authorship with one of the authors FD and state that the process nevertheless met the standards of a fair and objective review.”

The authors apologize for this error and state that this does not change the scientific conclusions of the article in any way. The original article has been updated.

## Publisher's Note

All claims expressed in this article are solely those of the authors and do not necessarily represent those of their affiliated organizations, or those of the publisher, the editors and the reviewers. Any product that may be evaluated in this article, or claim that may be made by its manufacturer, is not guaranteed or endorsed by the publisher.

