# Supplementary material for: Data-Driven Surveillance: Effective Collection, Integration, and Interpretation of Data to Support Decision Making
Source: Front Vet Sci. 2021 Mar 12;8:633977. doi: 10.3389/fvets.2021.633977 (PMC7994248; doi:10.3389/fvets.2021.633977)
Supplement: Supplementary file 1 [file Data_Sheet_1.docx]

Data-driven surveillance: Effective collection, integration and interpretation of data to support decision-making

Fernanda C. Dórea, Crawford W. Revie

Supplementary Material

We searched Scopus for papers published up to December 2020 in the general area of health surveillance which contained the term “big data” (TITLE-ABS-KEY ("big data" AND surveillance AND [health OR disease OR syndromic]). The search returned 492 papers. After reviewing title and abstract, and reading selected papers for which full-text was available in English, we selected a total of 44 papers which specifically discuss data science and data innovation challenges and opportunities in any area of health surveillance.

These 44 papers are listed below in alphabetical order (by first author’s last name):

Aiello, A.E., Renson, A., Zivich, P.N., 2019. Social media- and internet-based disease surveillance for public health. Annu. Rev. Public Health. https://doi.org/10.1146/annurev-publhealth-040119-094402

Antoine-Moussiaux, N., Vandenberg, O., Kozlakidis, Z., Aenishaenslin, C., Peyre, M., Roche, M., Bonnet, P., Ravel, A., 2019. Valuing health surveillance as an information system: Interdisciplinary insights. Front. Public Heal. 7. https://doi.org/10.3389/fpubh.2019.00138

Asokan, G.V., Asokan, V., 2015. Leveraging “big data” to enhance the effectiveness of “one health” in an era of health informatics. J. Epidemiol. Glob. Health 5, 311–314. https://doi.org/10.1016/j.jegh.2015.02.001

Balicer, R.D., Luengo-Oroz, M., Cohen-Stavi, C., Loyola, E., Mantingh, F., Romanoff, L., Galea, G., 2018. Using big data for non-communicable disease surveillance. Lancet Diabetes Endocrinol. 6, 595–598. https://doi.org/10.1016/S2213-8587(17)30372-8

Bansal, S., Chowell, G., Simonsen, L., Vespignani, A., Viboud, C., 2016. Big data for infectious disease surveillance and modeling. J. Infect. Dis. 214. https://doi.org/10.1093/infdis/jiw400

Barrett, D., 2017. The potential for big data in animal disease surveillance in Ireland. Front. Vet. Sci. 4. https://doi.org/10.3389/fvets.2017.00150

Bate, A., Reynolds, R.F., Caubel, P., 2018. The hope, hype and reality of Big Data for pharmacovigilance. Ther. Adv. Drug Saf. 9, 5–11. https://doi.org/10.1177/2042098617736422

Blazes, D.L., Dowell, S.F., 2019. The role of disease surveillance in precision public health, in: Genomic and Precision Medicine: Infectious and Inflammatory Disease. pp. 257–265. https://doi.org/10.1016/B978-0-12-801496-7.00015-0

Bragazzi, N.L., Dai, H., Damiani, G., Behzadifar, M., Martini, M., Wu, J., 2020. How big data and artificial intelligence can help better manage the covid-19 pandemic. Int. J. Environ. Res. Public Health 17. https://doi.org/10.3390/ijerph17093176

Bu, D.D., Liu, S.H., Liu, B., Li, Y., 2020. Achieving Value in Population Health Big Data. J. Gen. Intern. Med. 35, 3342–3345. https://doi.org/10.1007/s11606-020-05869-0

Buckee, C., 2020. Improving epidemic surveillance and response: big data is dead, long live big data. Lancet Digit. Heal. 2, e218–e220. https://doi.org/10.1016/S2589-7500(20)30059-5

Charles-Smith, L.E., Reynolds, T.L., Cameron, M.A., Conway, M., Lau, E.H.Y., Olsen, J.M., Pavlin, J.A., Shigematsu, M., Streichert, L.C., Suda, K.J., Corley, C.D., 2015. Using social media for actionable disease surveillance and outbreak management: A systematic literature review. PLoS One 10, 1–20. https://doi.org/10.1371/journal.pone.0139701

Chiolero, A., Chiolero, A., Chiolero, A., Chiolero, A., Buckeridge, D., 2020. Glossary for public health surveillance in the age of data science. J. Epidemiol. Community Health 74, 612–616. https://doi.org/10.1136/jech-2018-211654

Davidson, M.W., Haim, D.A., Radin, J.M., 2015. Using Networks to Combine “Big Data” and Traditional Surveillance to Improve Influenza Predictions. Sci. Rep. 5, 8154. https://doi.org/10.1038/srep08154

Degeling, C., Carter, S.M., Van Oijen, A.M., McAnulty, J., Sintchenko, V., Braunack-Mayer, A., Yarwood, T., Johnson, J., Gilbert, G.L., 2020. Community perspectives on the benefits and risks of technologically enhanced communicable disease surveillance systems: A report on four community juries. BMC Med. Ethics 21. https://doi.org/10.1186/s12910-020-00474-6

Dolley, S., 2018. Big data’s role in precision public health. Front. Public Heal. 6. https://doi.org/10.3389/fpubh.2018.00068

Eckmanns, T., Füller, H., Roberts, S.L., 2019. Digital epidemiology and global health security; An interdisciplinary conversation Tim Eckmanns, Leon Hempel, Kate Polin, Klaus Scheuermann, Edward Velasco. Life Sci. Soc. Policy 15. https://doi.org/10.1186/s40504-019-0091-8

Flahault, A., Bar-Hen, A., Paragios, N., 2016. Public Health and Epidemiology Informatics. Yearb. Med. Inform. 240–246. https://doi.org/10.15265/iy-2016-021

Gamache, R., Kharrazi, H., Weiner, J.P., 2018. Public and Population Health Informatics: The Bridging of Big Data to Benefit Communities. Yearb. Med. Inform. 27, 199–206. https://doi.org/10.1055/s-0038-1667081

Garattini, C., Raffle, J., Aisyah, D.N., Sartain, F., Kozlakidis, Z., 2019. Big Data Analytics, Infectious Diseases and Associated Ethical Impacts. Philos. Technol. 32, 69–85. https://doi.org/10.1007/s13347-017-0278-y

Gittelman, S., Lange, V., Gotway Crawford, C.A., Okoro, C.A., Lieb, E., Dhingra, S.S., Trimarchi, E., 2015. A New Source of Data for Public Health Surveillance: Facebook Likes. J. Med. Internet Res. 17, e98. https://doi.org/10.2196/jmir.3970

Hay, S.I., George, D.B., Moyes, C.L., Brownstein, J.S., Flaxman, A., 2013. Big Data Opportunities for Global Infectious Disease Surveillance. PLoS Med. 10, e1001413. https://doi.org/10.1371/journal.pmed.1001413

Hoffman, S., Podgurski, A., 2013. Big Bad Data: Law, Public Health, and Biomedical Databases. J. Law, Med. Ethics 41, 56–60. https://doi.org/10.1111/jlme.12040

Huang, T., Lan, L., Fang, X., An, P., Min, J., Wang, F., 2015. Promises and Challenges of Big Data Computing in Health Sciences. Big Data Res. 2, 2–11. https://doi.org/10.1016/j.bdr.2015.02.002

Khoury, M.J., Engelgau, M., Chambers, D.A., Mensah, G.A., 2019. Beyond Public Health Genomics: Can Big Data and Predictive Analytics Deliver Precision Public Health? Public Health Genomics 21, 244–249. https://doi.org/10.1159/000501465

Kumar, A.T.K., Asamoah, D., Sharda, R., 2015. Can social media support public health? Demonstrating disease surveillance using big data analytics, in: 2015 Americas Conference on Information Systems, AMCIS 2015.

Larson, E.B., 2013. Building Trust in the Power of “Big Data” Research to Serve the Public Good. JAMA 309, 2443. https://doi.org/10.1001/jama.2013.5914

Manogaran, G., Lopez, D., 2017. Disease Surveillance System for Big Climate Data Processing and Dengue Transmission. Int. J. Ambient Comput. Intell. 8, 88–105. https://doi.org/10.4018/IJACI.2017040106

Mavragani, A., 2020. Infodemiology and infoveillance: Scoping review. J. Med. Internet Res. 22. https://doi.org/10.2196/16206

Milinovich, G.J., Magalhães, R.J.S., Hu, W., 2015. Role of big data in the early detection of Ebola and other emerging infectious diseases. Lancet Glob. Heal. 3, e20–e21. https://doi.org/10.1016/S2214-109X(14)70356-0

Mooney, S.J., Pejaver, V., 2018. Big Data in Public Health: Terminology, Machine Learning, and Privacy. Annu. Rev. Public Health. https://doi.org/10.1146/annurev-publhealth-040617-014208

O’Shea, J., O ’shea, J., O’Shea, J., 2017. Digital disease detection: A systematic review of event-based internet biosurveillance systems. Int. J. Med. Inform. 101, 15–22. https://doi.org/10.1016/j.ijmedinf.2017.01.019

Othman, M.K., Danuri, M.S.N.M.M.S.N.M., 2016. Proposed conceptual framework of Dengue Active Surveillance System (DASS) in Malaysia, in: 2016 International Conference on Information and Communication Technology (ICICTM). IEEE, pp. 90–96. https://doi.org/10.1109/ICICTM.2016.7890783

Ouyang, Z., Sargeant, J., Thomas, A., Wycherley, K., Ma, R., Esmaeilbeigi, R., Versluis, A., Stacey, D., Stone, E., Poljak, Z., Bernardo, T.M., 2019. A scoping review of “big data”, “informatics”, and “bioinformatics” in the animal health and veterinary medical literature. Anim. Heal. Res. Rev. 1–18. https://doi.org/10.1017/S1466252319000136

Pollett, S., Althouse, B.M., Forshey, B., Rutherford, G.W., Jarman, R.G., 2017. Internet-based biosurveillance methods for vector-borne diseases: Are they novel public health tools or just novelties? PLoS Negl. Trop. Dis. 11. https://doi.org/10.1371/journal.pntd.0005871

Pyne, S., Vullikanti, A.K.S., Marathe, M. V., 2015. Big Data Applications in Health Sciences and Epidemiology, in: Handbook of Statistics. pp. 171–202. https://doi.org/10.1016/B978-0-444-63492-4.00008-3

Roberts, S.L., 2019. Big data, algorithmic governmentality and the regulation of pandemic risk. Eur. J. Risk Regul. 10, 94–115. https://doi.org/10.1017/err.2019.6

Santillana, M., 2017. Editorial Commentary : Perspectives on the Future of Internet Search Engines and Biosurveillance Systems. Clin. Infect. Dis. 64, 42–43. https://doi.org/10.1093/cid/ciw660

Simonsen, L., Gog, J.R., Olson, D., Viboud, C., 2016. Infectious Disease Surveillance in the Big Data Era: Towards Faster and Locally Relevant Systems. J. Infect. Dis. 214, S380–S385. https://doi.org/10.1093/infdis/jiw376

Smith, G.E., Elliot, A.J., Lake, I., Edeghere, O., Morbey, R., Catchpole, M., Heymann, D.L., Hawker, J., Ibbotson, S., McCloskey, B., Pebody, R., Bains, A., Harcourt, S., Hughes, H., Lee, W., Loveridge, P., Smith, S., Soriano, A., 2019. Syndromic surveillance: Two decades experience of sustainable systems – Its people not just data! Epidemiol. Infect. 147. https://doi.org/10.1017/S0950268819000074

Trifirò, G., Sultana, J., Bate, A., 2018. From Big Data to Smart Data for Pharmacovigilance: The Role of Healthcare Databases and Other Emerging Sources. Drug Saf. 41, 143–149. https://doi.org/10.1007/s40264-017-0592-4

Vallmuur, K., Marucci-Wellman, H.R., Taylor, J.A., Lehto, M., Corns, H.L., Smith, G.S., 2016. Harnessing information from injury narratives in the “big data” era: understanding and applying machine learning for injury surveillance. Inj. Prev. 22, i34–i42. https://doi.org/10.1136/injuryprev-2015-041813

Wong, Z.S.Y., Zhou, J., Zhang, Q., 2019. Artificial Intelligence for infectious disease Big Data Analytics. Infect. Dis. Heal. 24, 44–48. https://doi.org/10.1016/j.idh.2018.10.002

Zhang, W., Ram, S., Burkart, M., Pengetnze, Y., 2016. Extracting signals from social media for chronic disease surveillance, in: DH 2016 - Proceedings of the 2016 Digital Health Conference. https://doi.org/10.1145/2896338.2896340
